# Spirituality but not Religiosity Is Associated with Better Health and Higher Life Satisfaction among Adolescents

**DOI:** 10.3390/ijerph15122781

**Published:** 2018-12-07

**Authors:** Zuzana Dankulincova Veselska, Ivo Jirasek, Pavel Veselsky, Miroslava Jiraskova, Irena Plevova, Peter Tavel, Andrea Madarasova Geckova

**Affiliations:** 1 Department of Health Psychology, Faculty of Medicine, PJ Safarik University in Kosice, Trieda SNP 1, 04011 Kosice, Slovakia; andrea.geckova@upjs.sk; 2 Department of Recreology, Faculty of Physical Culture, Palacky University Olomouc, Trida Miru 115, 771 11 Olomouc, Czech Republic; ivo.jirasek@upol.cz; 3 Department of Sociology, Andragogy and Cultural Anthropology, Faculty of Arts, Palacky University Olomouc, Katerinska 17, 772 00 Olomouc, Czech Republic; pavel.veselsky@upol.cz; 4 Reinforcement of the Expert Potential of Research Teams in the Area of Physical Activity Support at Faculty of Physical Culture, Palacky University Olomouc, Trida Miru 115, 771 11 Olomouc, Czech Republic; miroslava.jiraskova@upol.cz; 5 Department of Psychology and Psychopathology, Faculty of Education, Palacky University Olomouc, Zizkovo Namesti 5, 771 40 Olomouc, Czech Republic; irena.plevova@upol.cz; 6 Olomouc University Social Health Institute, Palacky University Olomouc, Univerzitni 22, 771 11 Olomouc, Czech Republic; peter.tavel@upol.cz

**Keywords:** spirituality, religiosity, self-rated health, health complaints, life satisfaction

## Abstract

Careful conceptualization and differentiation of both spirituality and religiosity is a necessary precondition for understanding the potential role they play in health, whether physical or mental. The aim of this study was to explore the associations of spirituality with self-rated health, health complaints, and life satisfaction of adolescents with the moderating role of religiosity. Data from the Health Behaviour in School-aged Children study conducted in 2014 in Slovakia were used. The final sample consisted of 658 adolescents (mean age = 15.37; 50.6% boys). Data regarding spirituality, religiosity, self-rated health, health complaints, and life satisfaction were obtained. Binary logistic models revealed spirituality to be associated with self-rated health, health complaints, and life satisfaction. A moderating role of religiosity was not confirmed. The presented findings indicate the need to distinguish between the concepts of religiosity and spirituality in connection with subjective health and life satisfaction.

## 1. Introduction

Adolescence is considered to be a period of life during which future health patterns for adulthood are being established [1]. It is characterized as a period of relatively good physical and mental health, high life satisfaction, and low mortality. Within this developmental perspective, poor health and low life satisfaction can have a significant impact on the fulfillment of developmental challenges associated with adolescence and can lead to several long-term negative consequences in adulthood [2]. An increasing body of literature recognizes the importance of spirituality and religiosity and their possible role in maintaining good physical and mental health [3,4,5]. 

Careful conceptualization and differentiation of both spirituality and religiosity is a necessary precondition for understanding the potential role they play in health, whether physical or mental [4,6]. The understanding of the spiritual dimension should be based on its difference from religiosity [7]. Even though there is the possibility of overlap between the two terms, religiosity might be seen as established practices within a society related to a higher power, with the frequency of church attendance as a possible indicator of religiosity. In comparison, spirituality is more abstractly oriented on values and beliefs and associated with the meaning and purpose of life [8]. As stated by Walker and Dixon [9], people might consider themselves as spiritual and at the same time not be formally engaged in different forms of religious practices [10]. To summarize, though religion might be considered as a manifestation of spirituality, being religious does not automatically ensure spirituality [11].

The relationship between spirituality and physical health has been studied in previous research mostly from two perspectives: Either in connection with specific chronic diseases [12,13] or in connection with subjective health [11]. Attention has also been given to the connection between spirituality and different aspects of mental health among university and college students [14,15], secondary school students [16], and children [17,18].

Previous research on religiosity in association with health, including self-rated health, has been conducted mostly among adults [19,20,21] or adolescents [22]. The effects of religious affiliation and participation on self-reported health have been demonstrated among older Africans [23], older Koreans [24], and European populations [25]. Religiosity has also been found to be associated with mental health outcomes, including life satisfaction in adult or adolescent populations [26,27].

However, most of the studies have focused on adult or adolescent populations, and there is an existing research gap in relation to early adolescence. Our study is one of the few papers looking for a better understanding of the association between experienced spirituality and religiosity with self-rated health and life satisfaction in this age group. 

Therefore, the aim of this study was to explore the associations of spirituality with self-rated health, health complaints, and life satisfaction of Slovak adolescents, with the possible moderating role of religiosity (church attendance and importance of faith) and adjusted for age and gender. A higher level of spirituality was assumed to be associated with better self-rated health, fewer health complaints, and higher life satisfaction. At the same time, a moderating effect of religiosity was expected on the association of spirituality with self-rated health, health complaints, and life satisfaction.

## 2. Materials and Methods

### 2.1. Participants and Procedure

Data from the Health Behaviour in School-aged Children (HBSC) study conducted in 2014 in Slovakia were used. Each country has to follow a standardized international research protocol to ensure consistency in the survey instruments, data collection, and processing. The questions are subject to validation studies and piloting at the national and international levels, with the outcomes of these studies often being published, e.g., [28]. To obtain a representative sample, a two-step sampling was performed. In the first step, 151 larger and smaller elementary schools located in rural as well as in urban areas from all regions of Slovakia were asked to participate. These were randomly selected from a list of all eligible schools in Slovakia obtained from the Slovak Institute of Information and Prognosis for Education. In the end, 130 schools took part in the survey (response rate: 86.1%). In the second step, data from 10,179 adolescents from the 5th to 9th grades (response rate: 78.8%) were obtained. 

Non-responses were caused mainly by school absence due to illness or other reasons and the refusal of parents or adolescent to be involved in this study. Respondents younger than 11 years and older than 15.9 years (929 respondents) and respondents with missing responses regarding the studied variables were excluded (1655 respondents), leading to a final sample of 7595 adolescents (mean age: 13.53 years; 48.1% boys). Only a subsample of 14- to 16-years old adolescents from the 7th, 8th, and 9th grades was asked questions on spirituality and religiosity. Two versions of the questionnaire, with different sets of optional measures, were created in order to cover all the topics of national interest and, at the same time, to make administration within regular class time possible taking the length of the questionnaire into account. In order to ascertain the representativeness of this sub-sample, random selection was used for the distribution of these two versions of the questionnaire within a class. As a result, the final sample comprised 683 adolescents, of which 658 (mean age: 15.37; 50.6% boys) responded to the two essential questions for this manuscript. 

The study was approved by the Ethics Committee of the Medical Faculty at the P. J. Safarik University in Kosice (No: 9/2012). Parents were informed about the study via the school administration and could opt out if they disagreed with their child’s participation. Respondents themselves were provided with the opportunity to opt out from data collection following consent from their parents, if they did not wish to participate in it. Participation in the study was fully voluntary and anonymous, with no explicit incentives provided for participation. Questionnaires were administered by trained research assistants in the absence of a teacher during regular class time. 

### 2.2. Measures

Self-rated health was measured using the single item “Would you say your health is…?” with the response categories “excellent”, “good”, “fair”, and “poor”, and response categories were dichotomized (“excellent” and “good” vs. “fair”, and “poor”) as recommended by the HBSC study methodology [2,29].

The HBSC-symptoms checklist (HBSC-SCL) assessed the occurrence of eight subjective physical and psychological health complaints: headache, stomachache, backache, feeling low, irritability and bad temper, feeling nervous, sleeping difficulties, and feeling dizzy. The response categories indicating how frequently during the last 6 months the symptoms had occurred are “rarely or never”, “about every month”, “about every week”, “more than once a week”, and “about every day”. Responses for specific health complaints were dichotomized (“rarely or never” and “about every month” vs. “about every week”, “more than once a week” and “about every day”). Recurrent multiple health complaints were also computed and subsequently dichotomized, with two or more complaints at least once a week considered as displaying noticeable subjective health complaints [30].

Life satisfaction was measured with the Cantril ladder [31]. Respondents were asked to evaluate their satisfaction with life on the scale from 0 to 10. The response categories were dichotomized as “low” (0–5) vs. “normal-high” life satisfaction (6–10), as recommended by the HBSC study methodology [2,29]. 

Religiosity was measured by two questions covering the frequency of church attendance and self-rated importance of faith [32]. For church attendance, the following question was used: “How often do you go to church or to religious sessions?” with responses “several times a week”/“approximately once a week”/“approximately once a month”/“a few times a year”/“never”. Church attendance at least once a week was considered as regular church attendance. For faith importance, the following questions were used: “How important would you say your religious faith is for your life?” with responses ranging on the 7-point scale from “not important at all” to “absolutely important”. A score of at least 5 was considered to be high faith importance.

Spirituality was measured using a spirituality scale based on Gomez and Fisher’s Spiritual Well-Being Questionnaire [33], consisting of 8 items asking respondents: “How important is it for you to…” “be kind to others”, “have meaning in life”, “have a connection with nature”, “meditate or pray”, etc. The response categories ranged from “not at all important” (1) to “very important” (5). The sum score for the overall scale was computed, with a higher score indicating higher spirituality. Cronbach’s alpha for the overall scale was 0.86 and 0.81 for the Slovak and Czech Republic, respectively [34].

The Family Affluence Scale was used as a measure of socioeconomic status. The scale consists of six items that self-report material affluence. The questions were: Does your family own a car, van or truck? (Responses: (0) No, (1) One, (2) Two or more); Do you have your own bedroom for yourself? ((0) No, (1) Yes); How many computers does your family own? ((0) None, (1) One, (2) Two, (3) More than two); How many bathrooms (rooms with a bath/shower or both) are in your home? ((0) None, (1) One, (2) Two, (3) More than two); Does your family have a dishwasher at home? ((0) No, (1) Yes); and how many times did you and your family travel out of Slovakia for a holiday/vacation last year? ((0) Not at all, (1) Once, (2) Twice, (3) More than twice). The responses to the items are given as specific values and calculated as an aggregated FAS index ranging from 0 to 13. A higher score indicates higher family affluence.

### 2.3. Statistical Analysis

Descriptive statistics were used in the first step to explore the studied variables separately for boys and girls (n, %, mean, SD) and for the whole sample. In the next step binary logistic regression models were used to explore the association between spirituality as an independent variable and self-rated health, health complaints, and life satisfaction as dependent variables. Model 1 explores the crude effect of spirituality on self-rated health, health complaints, and life satisfaction. In Model 2 and 3, the possible moderating role of religiosity was tested using the interaction between church attendance with spirituality and the importance of faith with spirituality. All models were adjusted for age, gender, and family affluence. The analyses were performed with SPSS Statistics version 20. 

## 3. Results

Table 1 shows the basic descriptive statistics of the studied variables in the whole sample as well as for boys and girls separately. 

Table 2 presents the findings from logistic regression models between spirituality and self-rated health, adjusted for age, gender, and family affluence. Spirituality was significantly associated with self-rated health, with lower spirituality increasing the probability of worse self-rated health. Interactions of spirituality with church attendance and faith importance were tested but were not found to be significant.

Table 3 presents findings from logistic regression models between spirituality and health complaints adjusted for age, gender, and family affluence. Spirituality was significantly associated with health complaints, with lower spirituality increasing the probability of more frequent health complaints. Interactions of spirituality with church attendance and faith importance were tested but were not found to be significant.

Table 4 presents findings from logistic regression models between spirituality and life satisfaction adjusted for age, gender, and family affluence. Spirituality was significantly associated with life satisfaction, with higher spirituality increasing the probability of higher life satisfaction. Interactions of spirituality with church attendance and faith importance were tested but were not found to be significant.

## 4. Discussion

The aim of this study was to explore the associations of spirituality with self-rated health, health complaints, and life satisfaction of Slovak adolescents, with the possible moderating role of religiosity (church attendance and importance of faith) and adjusted for age, gender, and family affluence. A higher level of spirituality was assumed to be associated with better self-rated health, fewer health complaints, and higher life satisfaction. At the same time, a moderating effect of religiosity was expected on the association of spirituality with self-rated health, health complaints, and life satisfaction.

Spirituality was significantly associated with self-rated health, health complaints, and life satisfaction, with higher spirituality increasing the probability of higher life satisfaction and decreasing the probability of worse health and more frequent health complaints. These findings are in line with previously published research from older age groups. Studies on older adolescents and young adults produced results indicating that individuals who are more spiritual (e.g., have daily spiritual experiences, feel a spiritual connection to a god) experience better health, fewer health complaints, and higher life satisfaction than those less spiritual [11,35,36,37]. Results from the presented study add knowledge to the less frequent empirical studies conducted on early adolescents, suggesting a similar pattern on the association between spirituality and self-rated health, health complaints, and life satisfaction even in this early life period. An explanation might be found in the theory of positive youth development and resilience theory, which suggests meaning in life and life purpose as a part of spirituality to contribute to a youth’s positive development and consequently better health and higher life satisfaction [38,39]. 

Religiosity was not found to have a connection with self-rated health, health complaints, or life satisfaction. Previous research focused on the association of religiosity and spirituality with indicators of health has yielded inconclusive findings. Most of the studies revealed better health outcomes among religious groups; however, some of them show no health benefits [40,41]. However, it is important to take into account the role of different definitions and measurements used in these studies, as they might influence their findings. Both spirituality and religiosity are multidimensional constructs, and inconsistencies in findings presented in studies might result from only one or two specific dimensions being covered [42].

In addition, no interaction was found between spirituality and religiosity either, which suggests that religiosity does not play the role of moderator. Thus, it seems that the concepts of spirituality and religiosity are more distinguished than expected. As has already been mentioned, people might consider themselves as spiritual and at the same time not engage formally in different forms of religious practices [9,10,11]. Similar findings were found by Lun and Bond [43], where life satisfaction was found to be associated with spirituality-related beliefs but not with religiosity-related practices. In addition, as argued by Pittau, Zelli, and Gelman [44], better self-reported health and life satisfaction are closely linked to the perceived ability of people to realize their best potential, and such perception is expected to be more strongly associated with people’s evaluation of their spirituality. 

The presented findings are part of the international HBSC study, and consistency with its methodology represents one of its major strengths. At the same time, several limitations are present, such as using only a single item for measuring life satisfaction and self-rated health. However, both measures are widely used and have been validated in previous studies [30,45]. Furthermore, the cross-sectional design of the study does not allow us to confirm the causal relationship between the explored variables.

## 5. Conclusions

Spirituality was significantly associated with self-rated health, health complaints, and life satisfaction, with higher spirituality increasing the probability of higher life satisfaction and decreasing the probability of worse health and more frequent health complaints. However, no moderating role of religiosity was confirmed, which indicates the need to distinguish between the theoretical concepts of religiosity and spirituality.

## Figures and Tables

**Table 1:** Descriptive statistics (Mean, SD; n, %) of indicators of health, life satisfaction, spirituality, and religiosity for the whole sample and for boys and girls separately (N = 658, HBSC data collection 2014).

	Whole Sample (N = 658)		Boys (N = 333)		Girls (N = 325)	
Self-rated health (n, %)						
excellent and good	563	86.6	295	90.2	268	83.0
fair and bad	87	13.4	32	9.8	55	17.0
Health complaints (n, %)						
2 and more, more than once a week	259	40.9	94	70.9	165	53.1
None or 1, more than once a week	375	59.1	229	29.1	146	46.9
Life satisfaction (n, %)						
6–10	501	76.7	274	83.0	227	70.3
0–5	152	23.3	56	17.0	96	29.7
Church attendance (n, %)						
regularly	240	36.5	108	32.5	132	40.6
irregularly	417	63.5	224	67.5	193	59.4
Importance of faith (n, %)						
important	265	40.5	124	37.6	141	43.5
not important	389	59.5	206	62.4	183	56.5
Spirituality (Mean, SD)	29.74	6.27	28.85	6.69	30.65	5.68

Note: SD = standard deviation.

**Table 2:** Logistic regression with ORs and 95% CI of the association between spirituality and self-rated health with a possible moderating role of religiosity (N = 658, HBSC data collection 2014).

	Model 1	Model 2	Model 3
	OR (95% CI)	OR (95% CI)	OR (95% CI)
Age	1.28 (0.72–2.27)	1.27 (0.71–2.27)	1.26 (0.71–2.25)
Gender			
boys	Ref.	Ref.	Ref.
girls	2.22 (1.39–3.56) ***	2.34 (1.46–3.75) ***	2.34 (1.45–3.77) ***
Family affluence	0.94 (0.86–1.03)	0.93 (0.85–1.02)	0.93 (0.85–1.02)
Spirituality	0.96 (0.92–0.99) *	0.96 (0.93–0.99) *	0.95 (0.91–0.99) *
Church attendance			
irregularly		Ref.	
regularly		0.74 (0.08–6.98)	
Importance of faith			
not important			Ref.
important			0.43 (0.04–4.30)
Church attendance * spirituality		0.99 (0.92–1.07)	
Faith importance * spirituality			1.02 (0.95–1.10)

Note: * *p* ˂ 0.05, *** *p* ˂ 0.001, Ref. = reference group.

**Table 3:** Logistic regression with ORs and 95% CI of the association between spirituality and health complaints with a possible moderating role of religiosity (N = 658, HBSC data collection 2014).

	Model 1	Model 2	Model 3
	OR (95% CI)	OR (95% CI)	OR (95% CI)
Age	1.81 (1.17–02.81) **	1.82 (1.17–2.82) **	1.81 (1.16–2.80) **
Gender			
boys	Ref.	Ref.	Ref.
girls	3.08 (2.21–4.29) ***	3.06 (2.20–4.27) ***	3.11 (2.23–4.34) ***
Family affluence	0.95 (0.88–1.02)	0.95 (0.89–1.02)	0.95 (0.89–1.02)
Spirituality	0.96 (0.94–0.99) **	0.96 (0.93–0.99) *	0.94 (0.91–0.98) ***
Church attendance			
irregularly		Ref.	
regularly		1.14 (0.22–6.03)	
Importance of faith			
not important			Ref.
important			0.56 (0.10–3.14)
Church attendance * spirituality		0.99 (0.95–1.05)	
Faith importance * spirituality			1.03 (0.97–1.09)

Note: * *p* ˂ 0.05, ** *p* ˂ 0.01, *** *p* ˂ 0.001, Ref. = reference group.

**Table 4:** Logistic regression with ORs and 95% CI of the association between spirituality and life satisfaction with a possible moderating role of religiosity (N = 658, HBSC data collection 2014).

	Model 1	Model 2	Model 3
	OR (95% CI)	OR (95% CI)	OR (95% CI)
Age	0.92 (0.57–1.50)	0.93 (0.57–1.52)	0.94 (0.58–1.53)
Gender			
boys	Ref.	Ref.	Ref.
girls	0.43 (0.29–0.63) ***	0.41 (0.28–0.60) ***	0.43 (0.29–0.63) ***
Family affluence	1.15 (1.07–1.24) ***	1.16 (1.07–1.25) ***	1.15 (1.06–1.24) ***
			
Spirituality	1.04 (1.01–1.08) **	1.05 (1.01–1.09) **	1.05 (1.01–1.10) **
Church attendance			
irregularly		Ref.	
regularly		3.97 (0.59–26.55)	
Importance of faith			
not important			Ref.
important			2.46 (0.37–16.45)
Church attendance * spirituality		0.97 (0.91–1.03)	
Faith importance * spirituality			0.97 (0.92–1.04)

Note: * *p* ˂ 0.05, ** *p* ˂ 0.01, *** *p* ˂ 0.001, Ref. = reference group.

## References

[1] Sawyer S.M., Afifi R.A., Bearinger L.H., Blakemore S.J., Dick B., Ezeh A.C., Patton G.C. (2012). Adolescence: A foundation for future health. Lancet.

[2] Currie C., Zanotti C., Morgan A., Currie D., de Looze M., Roberts C., Samdal O., Smith O.R.F., Barnekow V. (2012). Social Determinants of Health and Well-Being among Young People. International Report from the HBSC 2009/2010 Survey. Health Policy for Children and Adolescents, No. 6.

[3] Aldwin C.M., Park C.L., Jeong Y.J., Nath R. (2014). Differing pathways between religiousness, spirituality, and health: A self-regulation perspective. Psychol. Relig. Spirit..

[4] Hill P.C., Pargament K.I. (2003). Advances in the conceptualization and measurement of religion and spirituality: Implications for physical and mental health research. Am. Psychol..

[5] Weber S.R., Pargament K.I. (2014). The role of religion and spirituality in mental health. Curr. Opin. Psychiatry.

[6] Miller W.R., Thoresen C.E. (2003). Spirituality, religion, and health: An emerging research field. Am. Psychol..

[7] Zinnbauer B.J., Pargament K.I., Cole B., Rye M.S., Butter E.M., Belavich T.G., Hipp K.M., Scott A.B., Kadar J.L. (1997). Religion and spirituality: Unfuzzying the fuzzy. J. Sci. Stud. Relig..

[8] Henningsgaard J.M., Arnau R.C. (2008). Relationships between religiosity, spirituality, and personality: A multivariate analysis. Personal. Individ. Differ..

[9] Walker K.L., Dixon V. (2002). Spirituality and academic performance among African American college students. J. Black Psychol..

[10] McSherry W., Cash K. (2004). The language of spirituality: An emerging taxonomy. Int. J. Nurs. Stud..

[11] Zullig K.J., Ward R.M., Horn T. (2006). The association between perceived spirituality, religiosity, and life satisfaction: The mediating role of self-rated health. Soc. Indic. Res..

[12] Parsian N., Dunning T. (2009). Developing and validating a questionnaire to measure spirituality: A psychometric process. Glob. J. Health Sci..

[13] Bekelman D.B., Dy S.M., Becker D.M., Wittstein I.S., Hendricks D.E., Yamashita T.E., Gottlieb S.H. (2007). Spiritual well-being and depression in patients with heart failure. J. Gen. Intern. Med..

[14] Jafari E., Dehshiri G.R., Eskandari H., Najafi M., Heshmati R., Hoseinifar J. (2010). Spiritual well-being and mental health in university students. Procedia Soc. Behav. Sci..

[15] Unterrainer H.F., Nelson O., Collicutt J., Fink A. (2012). The English version of the Multidimensional Inventory for Religious/Spiritual Well-Being (MI-RSWB-E): First results from British College students. Religions.

[16] Fisher J.W., Ryan J., Wittwer V., Baird P. (1999). Developing a Spiritual Health and Life-Orientation Measure for secondary school students. Research with a Regional/Rural Focus, Proceedings of the University of Ballarat Inaugural Annual Conference.

[17] Houskamp B.M., Fisher L.A., Stuber M.L. (2004). Spirituality in children and adolescents: Research findings and implications for clinicians and researchers. Child Adolesc. Psychiatr. Clin. N. Am..

[18] Stuber M.L., Houskamp B.M. (2004). Spirituality in children confronting death. Child Adolesc. Psychiatr. Clin. N. Am..

[19] Fisher K.A., Newbold K.B., Eyles J.D., Elliott S.J. (2013). Physical health in a Canadian Old Order Mennonite community. Rural Remote Health.

[20] Hunter B.D., Merrill R.M. (2013). Religious orientation and health among active older adults in the United States. J. Relig. Health.

[21] Merrill R.M., Steffen P., Hunter B.D. (2012). A Comparison of religious orientation and health between whites and Hispanics. J. Relig. Health.

[22] Powell-Young Y.M. (2012). Household income and spiritual well-being but not body mass index as determinants of poor self-rated health among African American adolescents. Res. Nurs. Health.

[23] Kodzi I.A., Gyimah S.O., Emina J., Ezeh A. (2011). Religious involvement, social engagement, and subjective health status of older residents of informal neighborhoods of Nairobi. J. Urban Health.

[24] You K.S., Lee H.O., Fitzpatrick J.J., Kim S., Marui E., Lee J.S., Cook P. (2009). Spirituality, depression, living alone, and perceived health among Korean older adults in the community. Arch. Psychiatr. Nurs..

[25] Nicholson A., Rose R., Bobak M. (2010). Associations between different dimensions of religious involvement and self-rated health in diverse European populations. Health Psychol..

[26] AbdAleati N.S., Zaharim N.M., Mydin Y.O. (2014). Religiousness and mental health: Systematic review study. J. Relig. Health.

[27] Michaelson V., Robinson P., Pickett W. (2014). Participation in church or religious groups and its association with health: A national study of young Canadians. J. Relig. Health.

[28] Brindova D., Pavelka J., Ševčikova A., Žežula I., van Dijk J.P., Reijneveld S.A., Geckova A.M. (2014). How parents can affect excessive spending of time on screen-based activities. BMC Public Health.

[29] Ravens-Sieberer U., Torsheim T., Hetland J., Vollebergh W., Cavallo F., Jericek H., Erhart M. (2009). Subjective health, symptom load and quality of life of children and adolescents in Europe. Int. J. Public Health.

[30] Currie C., Gabhainn S.N., Godeau E., Roberts C., Smith R., Currie D., Pickett W., Richter M., Morgan A., Barnekow V. (2008). Inequalities in Young People’s Health: International Report from the HBSC 2005/06 Survey. WHO Policy Series: Health Policy for Children and Adolescents Issue 5.

[31] Cantril H. (1965). The Pattern of Human Concern.

[32] Pitel L., Geckova A.M., Kolarcik P., Halama P., Reijneveld S.A., van Dijk J.P. (2012). Gender differences in the relationship between religiosity and health-related behaviour among adolescents. J. Epidemiol. Community Health.

[33] Gomez R., Fisher J.W. (2003). Domains of spiritual well-being and development and validation of the spiritual well-being questionnaire. Personal. Individ. Differ..

[34] Dutkova K., Holubcikova J., Kravcova M., Babincak P., Tavel P., Geckova A.M. (2017). Is spiritual well-being among adolescents associated with a lower level of bullying behaviour? The mediating effect of perceived bullying behaviour of peers. J. Relig. Health.

[35] Kim S., Miles-Mason E., Kim C.Y., Esquivel G.B. (2013). Religiosity/spirituality and life satisfaction in Korean American adolescents. Psychol. Relig. Spirit..

[36] Kelley B., Miller L. (2007). Life satisfaction and spirituality in adolescents. Res. Soc. Sci. Study Relig..

[37] Ratner P.A., Johnson J.L., Jeffery B. (1998). Examining emotional, physical, social, and spiritual health as determinants of self-rated health status. Am. J. Health Promot.

[38] Ungar M. (2008). Resilience across cultures. Br. J. Soc. Work.

[39] Lerner R.M., Dowling E.M., Anderson P.M. (2003). Positive youth development: Thriving as the basis of personhood and civil society. Appl. Dev. Sci..

[40] Mishra S.K., Togneri E., Tripathi B., Trikamji B. (2015). Spirituality and Religiosity and Its Role in Health and Diseases. J. Relig. Health.

[41] Levin J. (2013). Religious behavior, health, and well-being among Israeli Jews: Findings from the European Social Survey. Psychol. Relig. Spirit..

[42] Hooker S.A., Masters K.S., Carey K.B. (2014). Multidimensional assessment of religiousness/spirituality and health behaviors in college students. Int. J. Psychol. Relig..

[43] Lun V.M.C., Bond M.H. (2013). Examining the relation of religion and spirituality to subjective well-being across national cultures. Psychol. Relig. Spirit..

[44] Pittau M.G., Zelli R., Gelman A. (2010). Economic disparities and life satisfaction in European regions. Soc. Indic. Res..

[45] Idler E.L., Benyamini Y. (1997). Self-rated health and mortality: A review of twenty-seven community studies. J. Health Soc. Behav..

